# Effect of Metal Lathe Waste Addition on the Mechanical and Thermal Properties of Concrete

**DOI:** 10.3390/ma14112760

**Published:** 2021-05-23

**Authors:** Marcin Małek, Marta Kadela, Michał Terpiłowski, Tomasz Szewczyk, Waldemar Łasica, Paweł Muzolf

**Affiliations:** 1 Faculty of Civil Engineering and Geodesy, Military University of Technology in Warsaw, ul. Gen. Sylwestra Kaliskiego 2, 00-908 Warsaw, Poland; marcin.malek@wat.edu.pl (M.M.); mic.terpilowski@gmail.com (M.T.); tomasz.szewczyk6@gmail.com (T.S.); waldemar.lasica@wat.edu.pl (W.Ł.); pawel.muzolf@wat.edu.pl (P.M.); 2 Building Research Institute (ITB), ul. Filtrowa 1, 00-611 Warsaw, Poland

**Keywords:** recycling, lathe waste, CNC machining, sustainable development, mix modification, workability, mechanical properties, thermal properties

## Abstract

The amount of steel chips generated by lathes and CNC machines is 1200 million tons per year, and they are difficult to recycle. The effect of adding steel chips without pre-cleaning (covered with production lubricants and cooling oils) on the properties of concrete was investigated. Steel waste was added as a replacement for fine aggregate in the amounts of 5%, 10% and 15% of the cement weight, which correspond with 1.1%, 2.2% and 3.3% mass of all ingredients and 0.33%, 0.66% and 0.99% volume of concrete mix, respectively. The slump cone, air content, pH value, density, compressive strength, tensile strength, tensile splitting strength, elastic modulus, Poisson’s ratio and thermal parameters were tested. It was observed that with the addition of lathe waste, the density decreased, but mechanical properties increased. With the addition of 5%, 10% and 15% metal chips, compressive strength increased by 13.9%, 20.8% and 36.3% respectively compared to plain concrete; flexural strength by 7.1%, 12.7% and 18.2%; and tensile splitting strength by 4.2%, 33.2% and 38.4%. Moreover, it was determined that with addition of steel chips, thermal diffusivity was reduced and specific heat capacity increased. With the addition of 15% metal chips, thermal diffusivity was 25.2% lower than in the reference sample, while specific heat was 23.0% higher. No effect was observed on thermal conductivity.

## 1. Introduction

In 2019, global steel production was estimated at 1869.9 Mt, an increase of 3.4% compared to 2018 [1,2]. The construction sector generates a huge demand for steel [3,4]. Due to the development of the construction industry, a further increase in steel production is forecasted. The widespread use of steel products in the industry results in energy consumption [5], CO_2_ emission [6,7] and production of steel waste in various amounts and sizes [8,9,10,11]. Some steel waste is recycled [12,13,14]. Metal chips are generated during the cutting, milling and turning process as a side effect of manufacturing elements with specific geometric dimensions and surface finish [15,16]. These wastes represent about 3–5% by weight of metal casting. Moreover, it was estimated that industrial lathes produce about 3–4 kg of chips per working day [17,18], and according to an ICI report, the amount of waste generated by lathes and CNC machines is up to 1200 Mt per year [19,20,21]. Due to contamination of the chip surface with oils or other coolants during the machining process, the storage of this waste has a negative impact on the environment, and its cleaning generates additional costs [22]. Because of this, as well as due to their elongated spiral shape, small size and surface contamination, the recycling of metal chips is difficult [23]. In addition, the generated chips can have different properties due to different types of materials being processed.

It is possible to use a variety of waste as concrete components [24,25]. In literature it can be found that metal chips used in concrete mix are classified by other scientists as a replacement for aggregate or steel fibers.

With the addition of steel chips as a replacement for fine and coarse aggregate at 22%, 33% and 44% of cement mass, Maanvit et al. [26] obtained 19%, 27% and 19% respective increases in compressive strength compared to plain concrete and 50, 100 and 75% respective increases in flexural strength. Ismail and Al.-Hashmi [27] obtained 13% and 17% increases in compressive strength with the addition of iron filings at 28% and 37% of cement mass as a replacement for fine aggregate compared to the base sample, a but slight decrease (approximately 2%) for a 19% addition. The tensile strength increased by 23%, 24% and 28% respectively. With an increase in the amount of the addition to 56%, 75% and 94% of cement mass, 5%, 8% and 22% respective increases in compressive strength and 9%, 28% and 41% respective increases in flexural strength compared to the base sample were determined [28]. Alwaeli and Nadziakiewicz [29] demonstrated that with the addition of steel waste at 68%, 136%, 203% and 271% of cement mass (25%, 50%, 75% and 100% of fine aggregate), compressive strength respectively increased by 24%, 30%, 43% and 50% in relation with plain concrete. An inverse result was demonstrated by Hemanth Tunga et al. [30], who for addition of steel waste as replacement fine aggregate at 11%, 17% and 22% of cement weight obtained decreases in compressive strength. However, they used a mix of metal and plastic chips.

The addition of steel waste as fibers has been tested by other researchers [31,32,33,34]. Kumaran et al. [31] reported 5%, 11% and 9% increases in compressive strength and 9%, 19% and 10% increases in flexural strength for concrete with fiber addition at 7%, 10% and 13% of cement mass compared to plain concrete. Gawatre et al. [32] obtained an increase in compressive strength (11%) with the addition of fiber up to 25% of cement mass, and then a decrease. Similar results were demonstrated by Dharmaraj [33]. The compressive strength increased up to 10% of shredded scrap iron addition and then decreased. The maximum compressive strength was 53%; however, they used fly ash. Equally high increases in compressive strength (40%, 51% and 62%) were determined by Seetharam et al. [19] for concrete with fiber additions of 10, 20 and 30% of cement weight. For similar amounts of fibers (31%, 48% and 65% of cement mass), Mohammed et al. [34] obtained only 7%, 12% and 15% higher compressive strength compared to the reference sample.

Based on the above, it can be concluded that the results for concretes with the addition of metal chips are very different, and in order to commonly use them, further research is required. Moreover, different scientists use varied classifications of steel chips (some as aggregate, some as dispersed fibers). Therefore, this study aimed to assess the effect of steel chips as a replacement for fine aggregate on the mechanical and thermal properties of concrete. Compared to other research, lathe chips were used without pre-cleaning (covered with production lubricants and cooling oils), which is new and aims to increase the use of waste materials from the production process in construction. Tests of mixed (consistence, air content and pH value) and hardened concrete with 5, 10 and 15 wt.% of steel chips (compressive, flexural and split tensile strength, modulus of elasticity and Poisson’s coefficient, thermal conductivity and diffusivity and specific conductivity) were carried out. Steel chips in the amounts of 5, 10 and 15 wt.% of the cement weight, which correspond with 1.1%, 2.2% and 3.3% of mass of all ingredients and 0.33%, 0.66% and 0.99% of volume of concrete mix, respectively, were used. The obtained results show that it is possible to efficiently and ecologically manage lathe waste in concrete while improving its mechanical parameters.

## 2. Materials

### 2.1. Specimen Preparation

The mix was prepared using the following materials: Portland cement, aggregate, tap water, admixture and steel waste.

#### 2.1.1. Cement

CEM I 42.5R Portland cement according to the EN 197:1:2011 [35] standard was used (Górażdże Cement Works, Opole, Poland). The properties [36] and chemical composition of the cement are shown in Table 1 and Table 2, respectively.

#### 2.1.2. Aggregate

**Figure 1:**
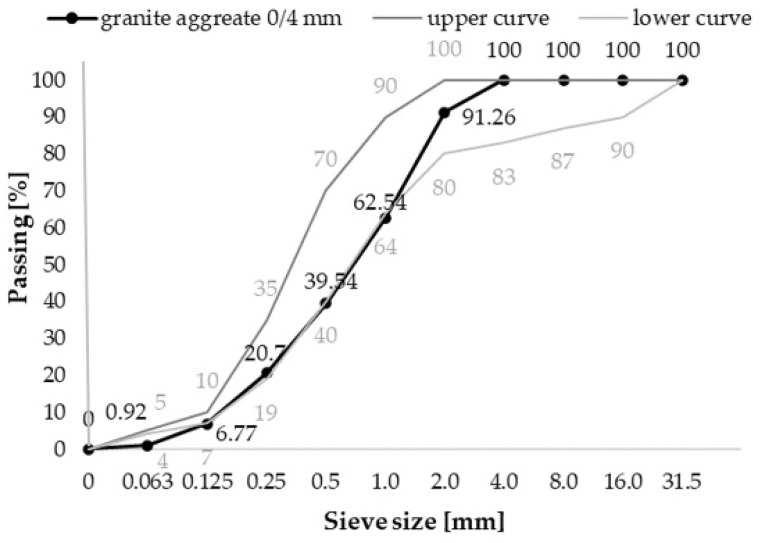
Particle size distribution curve for the used aggregate.

Crushed basalt sand with fractions of 0–4 mm was used as aggregate in the mixture, see Figure 1.

#### 2.1.3. Admixture

**Figure 2:**
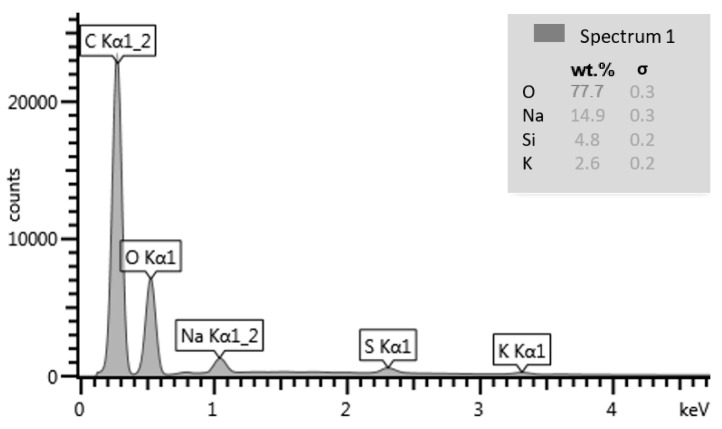
Chemical composition of admixture (%) [37].

Superplasticizer (Atlas Duruflow PE-531, Bydgoszcz, Poland) was used to reduce the amount of water required to liquefy the mixture. The product complies with the EN 934-2:2009+A1:2012 [38] standard. Figure 2 shows the chemical composition of the superplasticizer.

#### 2.1.4. Addition of Steel Waste

**Figure 3:**
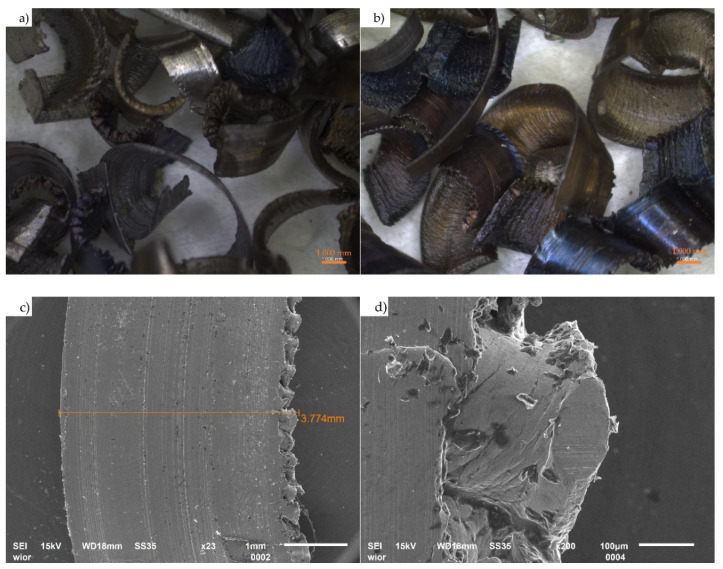
(**a**,**b**) Optical microscope images of steel chips used; (**c**,**d**) scanning electron microscope images of the steel chips’ surface (**c**) and edge (**d**).

Curved and slightly twisted steel chips (without large, stringy drill chips) of post-production origin generated by CNC machine tools were used (Figure 3). The chips were made from steel grade 18CrNiMo7-6 compliant with EN ISO 683-3:2019 [39]. This steel is used in the production of toothed wheels, gears and shafts. The waste is heterogeneous in terms of its material, shape, dimensions (Table 3) and degree of contamination. Its chemical composition, determined using XRF spectrometry, is shown in Table 4.

In this study, steel chips without pre-cleaning (covered with production lubricants and cooling oils) were used. Tests of organic substance content on the chip surface carried out at 450 °C showed the presence of organic substances of post-production origin in an amount of about 6%. By exposing the material to the temperature of 900 °C, the amount of organic substances increased to about 10% (Table 5). The increased loss on ignition (LOI) was probably due to the softening of the chips and the evaporation of their compounds.

### 2.2. Mixture Composition

Four types of mixtures were tested: a reference (without an additive of steel waste) and three mixes with different amounts of steel waste. Mixture composition is shown in Table 6. The composition of the reference mixture (M0) was established according to the method of designing the composition of the concrete recipe with an increased sand point, or sand concrete with a sand point value above 90%. The main idea behind the design of this type of concrete is the lack of coarse aggregate above 4.0 mm. The high proportion of fine-grained aggregate in the pile makes it necessary to cover the grains of the fine fractions with cement slurry, i.e., to use more cement (Table 5). Moreover, the strength of the concrete is mainly determined by the percentage of coarse-grained aggregate in the mixture composition. The designed (high) strength for concrete without coarse aggregate was obtained by adding a high cement content.

In the other mixtures (M1–M3, Table 6), the addition of steel waste was used as a fine aggregate replacement. The water–cement ratio for each mixture was 0.49.

### 2.3. Mixture Production

First, dry ingredients (the aggregate and steel chips) were mixed for 3 min. Then water mixed with a superplasticizer in the amount of 1% of cement weight was added. All components were mixed for 6 min, and then the mixture was poured into molds.

Molds in the shapes of cubes with dimensions of 150 mm × 150 mm × 150 mm; cylinders with dimensions of 150 mm × 300 mm and beams with dimensions of 100 mm × 100 mm × 500 mm and 40 mm × 40 mm × 160 mm were used. The mixture in the mold was compacted using a vibrating table. The top of the mold was prevented from water evaporation for 24 h after sample preparation. All samples were made in laboratory conditions (at 21 °C and 50% humidity). Samples were removed from molds after 36 h. The samples were stored in water until testing on day 28 in accordance with EN 12390-2:2019 [40].

## 3. Research Methodology

### 3.1. Testing the Concrete Mixtures

The consistency, air content and pH value of each concrete mixture were tested.

#### 3.1.1. Slump Test

**Figure 4:**
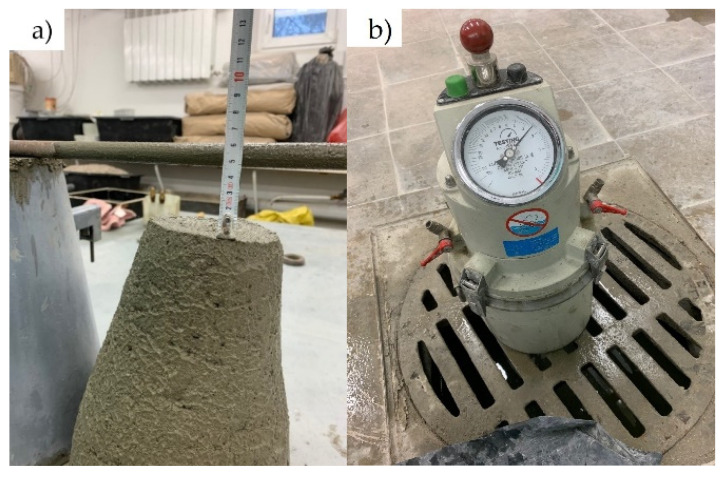
(**a**) Slump test, (**b**) measurement of air content (own photos).

The concrete mix consistency was determined using an Abrams cone (Merazet, Poznań, Poland) according to EN 12350-2:2019 [41] (Figure 4a). The slump cone test (SC) was carried out for five samples for each mixture.

#### 3.1.2. Air Content

Air content was tested using the pressure method according to EN 12350-7:2019 [42]. The sample was placed in an 8-liter container of porosimeter (Merazet, Poznan, Poland), and the vessel was filled with water under pressure. The measurement was recorded after pressure equalization (Figure 4b). The test was carried out for five samples for each mixture.

#### 3.1.3. PH Value

Acidity and alkalinity (pH) values were determined for the liquid phase extracted from fresh mixtures in accordance with PN-EN 1015-3:2001+A2:2007 [43]. The test was carried out using a Testo 206 ph2 device (Testo, Pruszków, Poland). The measured pH value was recorded after 180 s. The test was carried out for five samples for each mixture.

### 3.2. Test of Hardened Samples

The tests were carried out after 28 days of concrete hardening. Density and mechanical and thermal properties were determined for samples made from the analyzed mixtures (M0, M1, M2 and M3).

#### 3.2.1. Density

Density of hardened samples was determined for 150 mm × 150 mm × 150 mm cubes according to EN 12390-7:2019 [44]. The test was carried out for five samples for each mixture.

#### 3.2.2. Mechanical Properties

**Figure 5:**
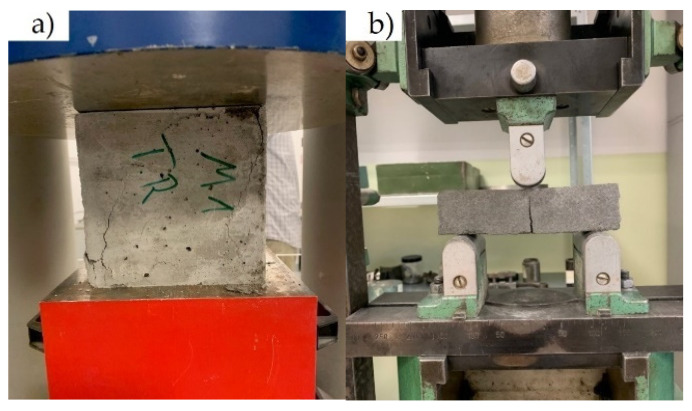
(**a**) Compressive and (**b**) flexural strength tests (own photos).

**Figure 6:**
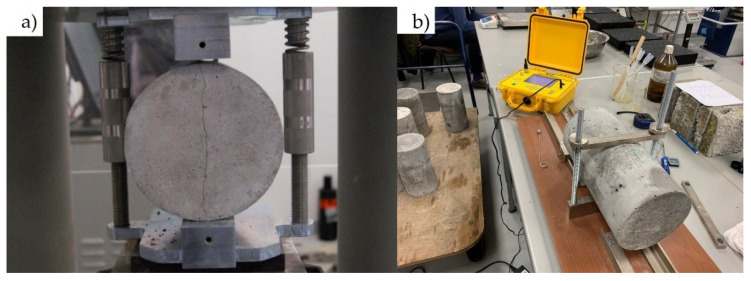
(**a**) Tensile splitting strength test and (**b**) measurement of Young’s modulus (own photos).

Tests were performed using a Zwick machine (Zwick, Ulm, Germany) with a force range of 0–5000 kN. Compressive strength was tested on 150 mm × 150 mm × 150 mm samples (cubes) according to EN 12390-3:2019 [45], see Figure 5a. Flexural strength was tested on 100 mm × 100 mm × 500 mm beams using a three-point bending system with support spacing of 300 mm (Figure 5b) according to EN 12390-5:2019 [46]. Splitting tensile strength was performed on 150 mm × 300 mm cylindrical samples according to EN 12390-6:2010 [47]—Figure 6a. Elastic modulus and Poisson’s ratio were tested on 150 mm × 300 mm cylindrical samples in accordance with EN 12390-13:2014 [48] by using 100 mm long strain gauges on two opposite sides of the specimens at half their height (Figure 6b). The edges of the compressive stressed samples were ground to avoid the effects of asymmetrical forces.

Each test was carried out for ten samples for each mixture.

#### 3.2.3. Thermal Properties

Thermal conductivity, thermal diffusivity and specific heat tests were carried out using the ISOMET2114 analyzer (Applied Precision Ltd., Bratislava, Slovakia). The test consisted in assessing the temperature change of the tested material per heat flow impulses. The heat was generated by electric resistor heaters on the probe with a diameter of 60 mm, which was in direct contact with the concrete sample with a minimum thickness of 25 mm. It was assumed that heat propagation takes place in an unrestricted body. The temperature was recorded as a function of time. Five measurements were taken at different locations for each sample. The average of the resulting readings was taken as the final result. The test was carried out on ten samples for each mixture.

## 4. Results

### 4.1. Concrete Mixture

The obtained results of the concrete mixture tests are shown in Table 7. The presented values are averages of five samples for each mixture. No particle agglomeration during mixing and pouring was demonstrated for each mixture.

The mixture workability increased for mixtures M1 and M2 compared to the reference mixture (Table 7). This may be due to the use of a superplasticizer in this study, because slump can be increased by addition of chemical admixtures (e.g., superplasticizer) without changing the water–cement ratio [49].

The tested mixtures were S1 class (M0 and M3) and S2 class (M1 and M2). Shewalul [50] obtained the same class of workability for 42.5 concrete class with the addition of steel scraps in the amount of 15% of cement content (0.5% vol. of concrete), which is analogous to the content of steel waste in this study.

The air content of tested mixtures with the addition of steel chips (Table 7) increased (from 2.8% ± 0.1% to 3.2% ± 0.1%) in comparison to plain concrete (2.3% ± 0.1%). This is probably a result of the irregular shapes of the chips. Furthermore, a slight effect of adding steel chips on the pH value of the mixture was observed (Table 7). Based on this, it can be concluded that the addition of chips using steel bars will not affect chip corrosion and the potential reinforcement of concrete.

### 4.2. Hardened Concrete

The obtained results are presented in Table 8 and Table 9. These values are the averages of five samples for each mixture for density and ten samples for other properties. On the basis of the obtained results, the relationships between the mechanical/thermal properties and the content of the used addition of lathe steel chips were prepared (see Section 5).

## 5. Discussion

Lathe scraps are added to concrete as replacement for aggregate or as fiber. Depending on this, its addition can be calculated in relation either to the aggregate or to the cement (see Section 1). In order to compare the results, in this article, the content of lathe chips was related to cement content. This is a proprietary approach to the issue of lathe chips as a component of concrete mix. Discussion of the individual properties of the mixtures and hardened concrete with the addition of steel chips is presented below.

### 5.1. Slump Cone

**Figure 7:**
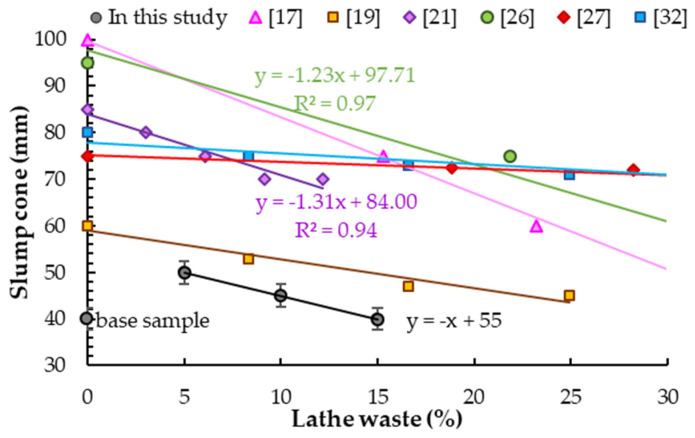
Slump cone test results.

With the addition of steel chips in the amount from 5% to 15% of cement mass, the workability of the mixture decreased (Figure 7). This trend is consistent with the observations of other scientists, regardless of use as aggregate replacement [26,27,28,51] or as dispersed fibers [17,19,21,32]. Prasad et al. [51] determined decreases in slump cone by 21%, 37% and 58% for steel waste up to 4.75 mm in length and up to 2 mm thick in the amount of 22%, 33% and 44% of the cement mass, respectively. The same results were obtained by Maanvit et al. [26] for steel waste with length of 10–20 mm, thickness of 0.25 mm and in the same amount. Ismail and Al.-Hashmi [28] also observed a 23% decrease in the slump cone for concrete with the addition of steel chips as a substitute for fine aggregate at 56% of cement weight, where 53% of chips with a size of 0.6–1.18 mm were used. While the same researchers obtained an 8% decrease for chips added at 38% of cement mass, in that case, 92% of the chips used were 1.18–2.36 mm in length [27]. A similar decrease in the slump cone with increasing fiber content compared to plain concrete was obtained by Gewatre et al. [32], who used steel chips as fiber in the range of 8–42% of cement weight. Seetharam et al. [19] reported more decreases in the slump cone for a concrete mixture with addition of lathe chips at 10%, 20% and 30% of cement weight. For 10% addition, the slump cone decreased by 12% compared to the reference mix. Abbas [17] determined a 25% decrease for steel fiber addition at 15.3% of cement weight and a 50% decrease for addition at 31.2% of cement weight due to poor anchoring of lathe chips. Purohit et al. [21] obtained a decrease in slump cone of 6%, 12%, 18% and 18% respectively for spiral steel chips 25–40 mm long and 0.3–0.75 mm thick at 3%, 6%, 9% and 12% of cement weight.

The less liquid consistency obtained in this study is due to the composition of the mixture. Moreover, it can be observed that for longer chips presented in this study and in [21,26], similar decreases in slump cone for the same steel chips content were determined (the slopes of the curves are similar (Figure 7)).

### 5.2. Density

**Figure 8:**
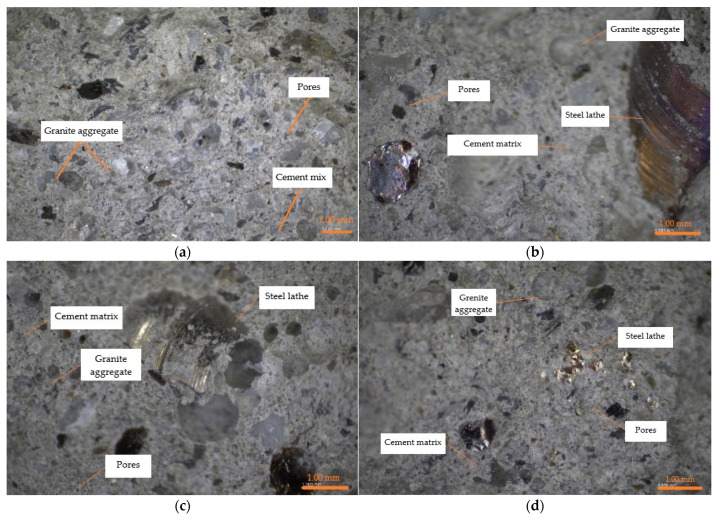
Concrete microstructure (OPTA-TECH, Warsaw, Poland): (**a**) M0, (**b**) M1, (**c**) M2, (**d**) M3.

With the increase in addition of steel waste (5%, 10% and 15% of cement mass), concrete density decreased by 2.9%, 7.0% and 8.6% compared to the reference sample, and this correlation is linear (Table 8). This was surprising because steel waste is heavier than granite aggregate. In order to explain this phenomenon, photographs of the concrete samples’ microstructure with different contents of steel chips were taken (Figure 8). It can be observed that there is no aggregation of steel chips and no air pockets. The chips are distributed uniformly and the sample is not segregated, which indicates that the test samples were made correctly. This phenomenon is probably related to the bulk density and that the aggregate did not fit in the places of the wound lathe chips. This will be the subject of further research.

**Figure 9:**
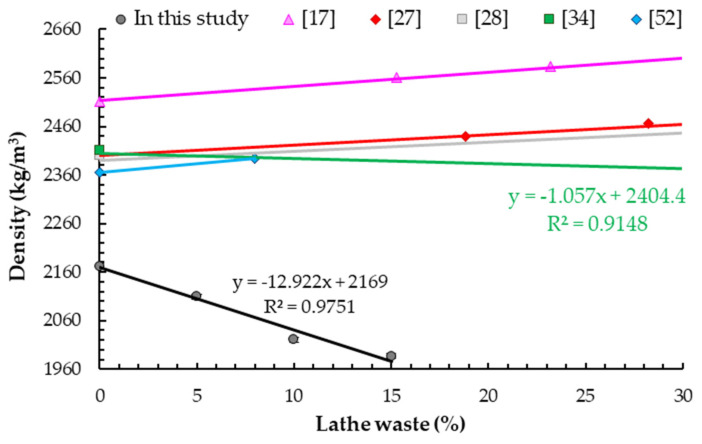
Density test results.

Moreover, similar observations were determined by Mohammed et al. [34], who obtained a 2.7% decrease in the density of the sample with addition of steel chips as fibers in the amount of 65% of cement weight compared to plain concrete (Figure 9). An inverse correlation was found by other researchers; however, the increase was insignificant (mostly less than 5% compared to plain concrete). Ismail and Al.-Hashmi [27] used steel chips, most of which (92%) were between 1.18 and 2.36 mm, as replacement fine aggregate, and they obtained a 3.2% increase in density for a sample with an addition of 37% of cement weight. Ismail and Al.-Hashmi [28] used chips, the majority (53%) of which were between 0.6 and 1.18 mm in length, and reported an 8% increase in density for concrete with an addition of steel waste at 94% of cement weight compared to a reference sample. Slight increases in density were determined by Abbas [17] and Qureshi and Ahmed [52], who used steel chips as fibers. Abbas [17], using steel chips (both straight and spiral, 50 mm in length, 1 mm in thick, 2 mm in wide), reported a 2% increase in density for concrete with an addition of chips at 15% of cement weight and 3.6% for concrete with an addition at 31% of cement weight. The same increase in density (1.9%) was obtained by Qureshi and Ahmed [52] for concrete with steel chips (up to 80 mm in size) at 52% of cement weight.

### 5.3. Compressive Strength

Table 8 shows the results of the compressive strength tests of the samples after 28 days of hardening. Compressive strength increased by 13.9% for M1, 20.8% for M2 and 36.3% for M3 in relation to the reference sample (50.4 ± 0.3 MPa). The same results were determined by Arunakanthi and Ch. Kumar [53] and Shewalul [50]. Arunakanthi and Ch. Kumar [53] obtained 15%, 22% and 25% increases in compressive strength for concrete with chips added at 6%, 12% and 19% of cement mass. Prabu et al. [54] demonstrated about a 20% improvement for 5% and 10% of cement mass used as fine aggregate replacement. Shewalul [50] achieved a 26.8% increase for steel chips addition at 13% of cement mass, which is similar to results obtained in this study. Moreover, increases in compressive strength for concrete with steel chips as a substitute for fine aggregate have been observed by other scientists. Shukla [20] obtained lower compressive strength increases (5 and 14% for the addition of chips in the amount at 6% and 12% of cement mass) than in this study. The same effect was demonstrated by Ismail and Al.-Hashmi [27,28], while a higher compressive strength increase (23%) was demonstrated by Hemanth Tunga et al. [30] for addition at 6% of cement mass. Sheikh and Reza [55] obtained a 35.9% improvement, but they used a lathe waste as coarse aggregate replacement. Alwaeli and Nadziakiewicz [29] determined higher increases in compressive strength (24%, 30%, 43% and 50%), but for much larger content of steel chips (68%, 136%, 203% and 271% of cement weight and 25%, 50%, 75% and 100% of fine aggregate).

The same trend was observed for lathe chips used as steel fibers [26,32,51]. Kumaran et al. [31] reported 5%, 11% and 9% increase in compressive strength for concrete with fiber addition in amounts analogous to this study (7%, 10% and 13% of cement mass) compared to plain concrete. Ghumare [56] observed 16.0%, 19.5% and 21.3% improvement for addition at 4%, %6 and 8% of cement mass and a w/c ratio equal to 0.4 (the calculated increase for 5% of cement mass was equal to 17.7%). Althoey and Hosen [57] obtained 5% and 13% increases for the same cement class as in this study and for metal chips added at 10.8% and 21.6% of cement mass, respectively.

A higher compressive strength increase (40%) was obtained by Seetharam et al. [19] for addition at 10% of cement mass, while Purohit et al. [21] demonstrated increases of 3%, 4%, 20% and 8% for steel chips added at 3%, 6%, 9% and 12% of cement weight respectively.

A similar increase in compressive strength in relation to the reference sample (15% and 13%) as in this study was observed by Mansi et al. [18] for addition of steel fibers at 1% and 2% of the sample weight. 11–13% increases were also observed by other scientists [58,59,60]. Ashok et al. [61] and Shrivastavaa and Joshib [62] obtained a maximum strength increase of about 3% for concrete with lower content of steel chips added (at 0.5–2% of cement weight).

**Figure 10:**
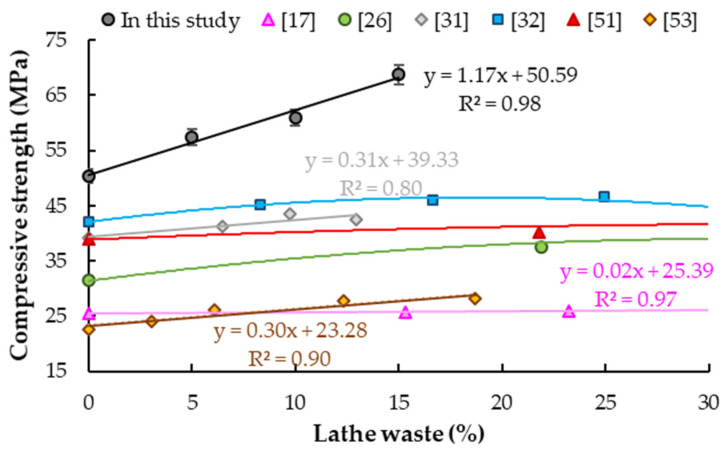
Results of compressive strength tests of concrete samples with different steel chip contents.

The samples with metal lathe waste addition were destroyed in the same way as the reference sample. The relation between compressive strength and the addition of chips is linear. The obtained results are in line with the observations of other scientists (Figure 10). For addition lathe chips as fibers at 7%, 10% and 13% of cement weight, Kumaran et al. [31] determined 5%, 11% and 9% increases in compressive strength in relation to the reference sample, but those values are almost half of those obtained in this study. Abbas [17] obtained a slight 2% increase in strength for the addition of lathe chips at 15%, 23% and 31% of cement weight in relation to the reference sample. For additives of 31%, 48% and 65% by weight of cement, Mohammed et al. [34] obtained compressive strength increases of 7%, 12% and 15% respectively compared to the reference sample. It can be observed that the obtained correlation between compressive strength and lathe waste content is similar to relationships determined by other scientists [17,31,53]. In this case the slope of the curve is an approximate (Figure 10).

Concrete with analogous added content to that in this study (6%, 11%, 17% and 22% of cement weight) was tested by Hemanth Tunga et al. [30], and they obtained a 23% increase in compressive strength for a 6% addition of chips, but then a decrease. The same correlation was determined by Gawatre et al. [32] for concrete with lathe chips added at 8–42% of cement weight, but the maximum increase in compressive strength (11%) was obtained for an addition of 25% of cement weight. The increase in compressive strength and then decrease was also observed by Maanvit et al. [26] and Prasad et al. [51]. Dharmaraj [33] also obtained an increase in compressive strength up to 53% for 10% of shredded scrap iron addition, and then a decrease.

An almost incredible increase in compressive strength with the increase in steel waste addition can be observed here, while the experimental studies of other scientists indicate a rather slight or even negligible increase. This may be related to the adopted mixture composition (see Section 2).

### 5.4. Flexural Strength

**Figure 11:**
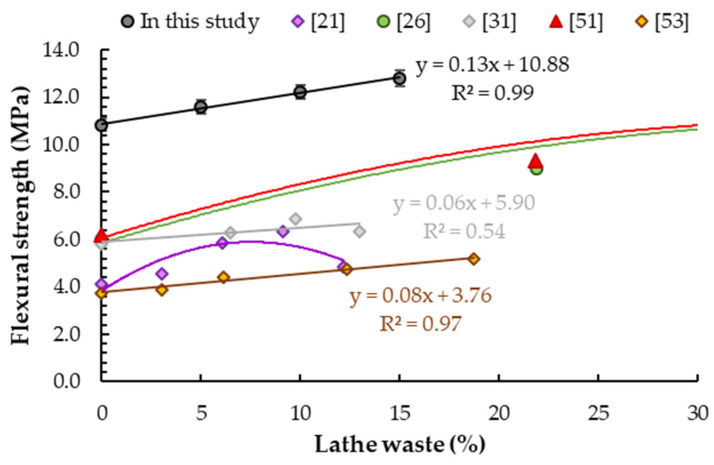
Results of tensile flexural strength tests of concrete samples with different steel chip contents.

The results of the flexural strength of the samples are shown in Table 8. It was observed that with the addition of steel lathe waste at 5%, 10% and 15% of cement weight, the increase in flexural strength was 7.1%, 12.7% and 18.2% respectively compared to the reference sample (10.8 ± 0.1 MPa). Figure 11 shows a linear relationship between the tensile flexural strength of the sample and the content of steel chip addition. The same conclusion was determined by Arunakanthi and Ch. Kumar [53]. They obtained higher increases in flexural strength (18%, 27% and 38%) compared to the base sample for the same content of steel chip addition (3%, 6%, 9% and 12%), but in this case, steel chips were used as fiber. However, if the amount of lathe chips is taken in relation to the cement content, it can be observed that correlation between flexural strength and lathe waste content is similar (the slope of the curves is an approximate (Figure 11)). The same correlation was determined by Kumaran et al. [31], who reported 9%, 19% and 10% increases in flexural strength for concrete with fiber addition at 7%, 10% and 13% of cement mass compared to plain concrete. This is related to the prevention of crack formation and the bridging of cracks analogously to the case of using traditional fibers [63,64,65] or recycled fibers [66,67,68]. This phenomenon for concrete with lathe chips as fiber was presented by Shrivastavaa and Joshi [62]. In this study, the steel chips were used as replacement of fine aggregate, so these tests were not analyzed.

The increase in flexural strength with increasing steel chip content is in line with the observations of other scientists (Figure 11). Similar improvement (13.15%) was observed by Ghumare [56] for concrete with lathe chip content at 8% of cement mass and a w/c ratio equal to 0.45, but the value was lower than the results obtained in this study. Prabu et al. [54] obtained similar values of flexural strength for M30 concrete with 5% and 10% addition of steel chips and w/c = 0.4, but the increase was much higher than in this study. Ismail and Al.-Hashmi [27] obtained a similar increase in flexural strength (22%) in relation to the base sample for steel chips added as replacement for fine aggregate at 19% of cement weight. In another test, Ismail and Al.-Hashmi [28] obtained a slightly lower increase in tensile strength (9%) for the same content of chips, but this was probably due to the use of a mix of metal and plastic chips in this case. Additions at 22%, 33% and 44% of cement weight were tested by Maanwit et al. [26], who obtained 50%, 100% and 75% increases in flexural strength in relation to the reference sample. Similar increases (50%, 94% and 69%) were obtained by Prasad et al. [51] for the same amounts of addition of lathe chips but at a greater size (as replacement for fine and coarse aggregate). Dharmaraj [33] studied concrete with the addition of fly ash and shredded scrap iron as steel fiber at 10–25% of cement weight and obtained an increase in compressive strength of up to 3% for 15% scrap iron. Ashok et al. [60] used a slight amount of steel chip addition in the range of 0.5–2% of cement weight and obtained a maximum increase in strength of 42% compared to the reference sample. A decrease in strength was presented by Seetharam et al. [19] for the addition of steel chips as fiber at 10%, 20% and 30% of cement weight.

### 5.5. Splitting Tensile Strength

**Figure 12:**
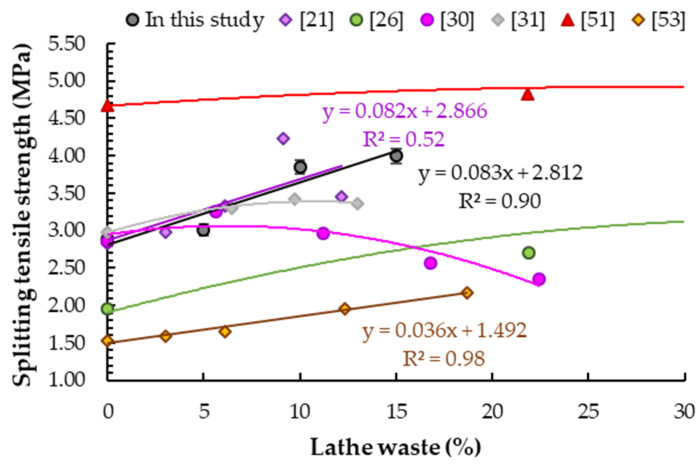
Results of tensile splitting strength tests of concrete samples with different steel chip contents.

Results of splitting tensile strength tests of samples with different steel chip contents are shown in Table 8. It was observed that with the addition of steel lathe waste at 5%, 10% and 15% of cement weight, the increase in splitting tensile strength was 4.2%, 33.2% and 38.4% respectively compared to the reference sample (2.89 ± 0.03 MPa). Prabu et al. [54] obtained the same values of flexural strength and the same increase for M30 concrete with 5% and 10% addition of steel chips and w/c = 0.4. Shewalul [50] achieved similar values (4.37 MPa) for M25 concrete with steel chips used as fine aggregate replacement at 13% of cement mass, but the increase was much lower (11.2%). Increases in splitting tensile strength compared to base samples were also observed by other scientists (Figure 12). Kumaran et al. [31] reported lower increases in splitting tensile strength (11%, 15% and 13%) compared to the reference sample for the same addition as in this study (7%, 10% and 13% of cement weight). Hemanth Tunga et al. [30] obtained the highest increase in splitting tensile strength (14%) for a sample with 6% steel chips addition of cement mass, and then a decrease. Maanvit et al. [26] obtained increases in strength of 38%, 79% and 51% in relation to the reference sample for samples with addition of steel chips at 22%, 33% and 44% of cement weight respectively. Moreover, Prasad et al. [51], for the same amounts of addition, but for a mix of steel and plastic chips, determined much lower increases in tensile split strength at 3%, 7% and 3%. The same correlation was observed by Mohammed et al. [34] and Kumaran et al. [31]. Mohammed et al. [34] obtained splitting tensile strength increases of 42%, 11% and 20% for concrete with additions of 31%, 48% and 65% by weight of cement, respectively. Ashok et al. [61] tested a lower amount of additive in the range of 0.5–2.0% of cement weight, and they obtained the maximum increase in splitting tensile strength at 20%.

Analogously to other mechanical properties, it can be observed that the slope of the obtained curve for correlation between splitting tensile strength and lathe waste content is very similar to relationships determined by other scientists [21,53], especially to the results obtained by Purohit et al. [21] (Figure 12).

### 5.6. Elastic Modulus and Poisson’s Ratio

With the addition of steel waste, the elasticity modulus increased from 0.6% to 6.2% compared to plain concrete (32.0 ± 0.4 GPa) (Table 8). This phenomenon is consistent with results obtained by Shewalul [50], but values demonstrated in this study were higher.

Poisson’s ratio for the reference sample was 0.120 ± 0.03. It can be observed that the results for samples M1–M3 were within the measurement error limit (Table 8).

### 5.7. Thermal Properties

Thermal parameters for concrete samples are presented in Table 9. The addition of lathe waste (between 5% and 15% of cement mass) did not affect thermal conductivity.

**Figure 13:**
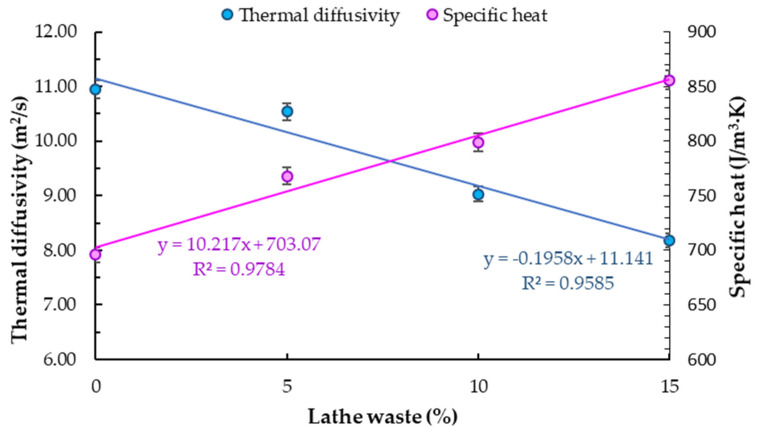
Specific heat and thermal diffusivity of concrete.

For a 5%, 10% and 15% addition of lathe waste, a 3.7%, 17.5% and 25.2% decrease in thermal diffusivity was obtained in relation to the reference sample. A linear correlation between steel chip content and thermal diffusivity was demonstrated (Figure 13). In addition, Figure 13 shows specific heat results for concrete with different amounts of steel chips. With the addition of 5%, 10% and 15% of lathe waste, an increase in specific heat by 10.4%, 14.8% and 23.0% respectively was obtained compared to the base sample. The obtained correlation is similar to concrete with steel fibers [14].

## 6. Conclusions

The aim of the study was to assess the possibility of using post-production waste materials in the form of lathe chips without pre-cleaning (covered with production lubricants and cooling oils) in concrete. In this study, three different contents of addition (5%, 10% and 15% of cement weight) were used as substitutes for fine aggregate. The following conclusions can be drawn on the basis of this experimental study’s results:

1. For a waste addition of 5% and 10% of cement weight, the workability of the concrete mix increased and was qualified to the S2 class. The reference samples and the samples with additions of 15% waste lathe were S1 class.
2. The air content of tested mixtures with the addition of steel chips (Table 7) increased (from 2.8% ± 0.1% to 3.2% ± 0.1%) in comparison to plain concrete (2.3% ± 0.1%). This is probably a result of the irregular shape of chips.
3. The compressive strength of concrete after 28 days increased linearly from 50.4 MPa to 68.7 MPa in relation to the reference sample. For an addition of steel chips at 15% of cement weight, a 36.3% increase in compressive strength was obtained.
4. The flexural strength increased linearly from the reference value of 10.83 MPa to 12.8 MPa. This corresponds to an 18.2% increase in flexural strength for the 15 wt.% addition of steel chips.
5. The splitting tensile strength for the 15 wt.% additive increased by 38.4% compared to the reference sample.
6. A slight increase in the elastic modulus from about 1% to 6% was observed for additions of metal lathe waste from 5% to 15% of cement weight.
7. It was demonstrated that the addition of steel chips as a substitute for fine aggregate does not affect the thermal conductivity of concrete.
8. A 3.7%, 17.5% and 25.2% decrease in thermal diffusivity was obtained for the addition of steel chips at 5%, 10% and 15% of cement weight.
9. The specific heat for concrete with addition of 15% steel chips as a replacement for fine aggregate was higher by 23.0% compared to the reference sample.

Moreover, in this study it was determined that thanks the adoption of lathe chip content in relation to the cement content, correlations between properties and lathe chip content for different concrete mixes are similar. The slopes of the curves are approximate.

Based on the obtained results and correlations between properties and lathe chip content, differences can be observed between these results and results obtained by other scientists due to the use of different cement classes, different aggregate type and fraction, type of plasticizers and the type, quality and level of contamination of the added steel chips. Moreover, it was determined that use of lathe chips without pre-cleaning (covered with production lubricants and cooling oils) as replacement for fine aggregate in the amount of 5% to 15% can improve the mechanical properties of concrete. It was particularly surprising that better results were obtained than with chips processed using the classical method entailing their melting.

This paper is part of a wider research project aimed at developing environmentally friendly concrete in accordance with the principles of sustainable development and closed-loop economy by using recycled construction materials in concrete. Taking into account the improvements in the mechanical properties of concrete through the addition of chips, further testing of this material, especially fatigue testing, will be scheduled.

## Figures and Tables

**Table 1:** Physical properties of cement [36].

Specific Surface Area (m^2^/kg)	Specific Gravity (kg/m^3^)	Compressive Strength after Days (MPa)	
		2 Days	28 Days
3.874	3050–3140	27.8	59.3

**Table 2:** Chemical composition of cement [37].

Compositions	SiO_2_	Al_2_O_3_	Fe_2_O_3_	CaO	MgO	SO_3_	Na_2_O	K_2_O	Cl
Unit (vol. %)	19.5	4.9	2.9	63.3	1.3	2.8	0.1	0.9	0.05

**Table 3:** Chip dimensions.

Description	Length (mm)	Width (mm)	Thickness (mm)
Dimension range	8.8–16.8	3.8–4.6	0.237–0.244
Mean value	12.3 ± 0.5	4.1 ± 0.5	0.240 ± 0.02

**Table 4:** Chemical composition, XRF results.

Compositions	Fe	C	Mn	Si	P	S	Cr	Ni	Mo	W	V	Cu
Unit (vol.%)	95.38	0.18	0.5	0.32	0.016	0.020	1.5	1.5	0.26	0.02	0.1	0.2

**Table 5:** Results of loss on the ignition methods at 450 **°C** and 900 **°C**.

No.	Initial Weight (g)	Final Weight (g)	LOI (%)
1 (450 °C)	17.385	16.287	6.32
2 (450 °C)	19.976	18.596	6.91
3 (450 °C)	15.541	14.533	6.49
**Average**	**-**	**-**	**6.57**
1 (900 °C)	20.219	17.947	11.24
2 (900 °C)	18.935	16.913	10.68
3 (900 °C)	19.541	17.499	10.45
**Average**	**-**	**-**	**10.79**

**Table 6:** Mix proportions (1 m^3^).

Mix Symbol	Cement (kg)	Water (kg)	Admixture (kg)	Aggregate (kg)	Lathe Waste (wt.% of Cement)	Lathe Waste (kg)
M0	511	250	0.51	1535.00	0	0.00
M1				1526.50	5	25.55
M2				1518.01	10	51.10
M3				1509.52	15	76.65

**Table 7:** Results of the slump test, air content and pH value tests.

Mix Symbol	Lathe Waste			Slump Cone (mm)	Air Content (%)	pH Value (-)
	(wt.% of Cement)	(wt.% of Component)	(vol.% of Mix)			
M0	0	0	0	40 ± 1	2.3 ± 0.1	12.02 ± 0.03
M1	5	1.1	0.33	50 ± 2	2.8 ± 0.1	12.09 ± 0.03
M2	10	2.2	0.66	45 ± 1	3.1 ± 0.1	12.16 ± 0.04
M3	15	3.3	0.99	40 ± 2	3.2 ± 0.1	12.20 ± 0.03

**Table 8:** Experimental results of material and mechanical properties.

Mix Symbol	Lathe Waste			Density (kg/m^3^)	Compressive Strength (MPa)	Flexural Strength (MPa)	Splitting Tensile Strength (MPa)	Young’s Modulus (GPa)	Poisson’s Ratio (-)
	(wt.% of Cement)	(wt.% of Component)	(vol.% of Mix)						
M0	0	0	0	2172 ± 2	50.4 ± 0.3	10.8 ± 0.1	2.89 ± 0.03	32.0 ± 0.4	0.120 ± 0.03
M1	5	1.1	0.33	2109 ± 2	57.4 ± 0.7	11.6 ± 0.1	3.01 ± 0.05	32.2 ± 0.3	0.121 ± 0.04
M2	10	2.2	0.66	2021 ± 3	60.9 ± 0.5	12.2 ± 0.1	2.85 ± 0.03	33.2 ± 0.4	0.123 ± 0.03
M3	15	3.3	0.99	1986 ± 2	68.7 ± 0.7	12.8 ± 0.1	4.00 ± 0.04	34.0 ± 0.3	0.123 ± 0.04

**Table 9:** Experimental results of thermal properties.

Mix Symbol	Lathe Waste			Thermal Conductivity (W/m∙K)	Thermal Diffusivity ×10^6^ (m^2^/s)	Specific Heat (J/m^3^∙K)
	(wt.% of Cement)	(wt.% of Component)	(vol. % of Mix)			
M0	0	0	0	1.6 ± 0.1	1.09 ± 0.07	696 ± 4
M1	5	1.1	0.33	1.6 ± 0.1	1.05 ± 0.07	768 ± 4
M2	10	2.2	0.66	1.4 ± 0.2	0.90 ± 0.07	799 ± 4
M3	15	3.3	0.99	1.4 ± 0.3	0.82 ± 0.07	856 ± 4

## Data Availability

Not applicable.
